# Crude extract from *Libidibia ferrea* (Mart. ex. Tul.) L.P. Queiroz leaves decreased intra articular inflammation induced by zymosan in rats

**DOI:** 10.1186/s12906-019-2454-3

**Published:** 2019-02-12

**Authors:** Tamires Rocha Falcão, Cássio Alexandre Oliveira Rodrigues, Aurigena Antunes de Araújo, Caroline Addison Carvalho Xavier de Medeiros, Luiz Alberto Lira Soares, Magda Rhayanny Assunção Ferreira, Roseane Carvalho Vasconcelos, Raimundo Fernandes de Araújo Júnior, Maria Luiza Diniz de Sousa Lopes, Gerlane Coelho Bernardo Guerra

**Affiliations:** 1 Postgraduate program in Pharmaceutical Science / Department of Biophysics and Pharmacology, UFRN, Natal, RN Brazil; 2 Department of Biophysics and Pharmacology, UFRN, Natal, RN Brazil; 3 Postgraduate program in Oral Science / Postgraduate program in Pharmaceutical Science/ Department of Biophysics and Pharmacology, Centro de Biociências/UFRN, Av. Senador Salgado Filho, S/N, Campus Universitário, Lagoa Nova, 59072-970, Natal, RN Brazil; 4 Postgraduate in Biological Science / Postgraduate program in RENORBIO /Department of Biophysics and Pharmacology, UFRN, Natal, RN Brazil; 5 Laboratory of Pharmacognosy / Postgraduate program in Therapeutic Innovation/ Department of Pharmaceutical Sciences, UFPE, Recife, PE Brazil; 6 Postdoctoral in the Postgraduate program in Public Health. Department of Dentistry, UFRN, Natal, RN Brazil; 7 Post graduation program in Functional and Structural Biology/Post graduation program Health Science/Department of Morphology, UFRN, Natal, RN Brazil; 8 Post Graduation in Biological Science/Post graduation program in Pharmaceutical Science/Department of Biophysics and Pharmacology, UFRN, Natal, RN Brazil

**Keywords:** *Libidibia ferrea*, Intra-articular, Inflammation, Oxidative stress

## Abstract

**Background:**

*Libidibia ferrea* (*L. ferrea)* has been used in folk medicine to treat several conditions and to prevent cancer. This study performed a chromatographic analysis of the crude aqueous extract of *Libidibia ferrea (Mart. ex. Tul.) L.P. Queiroz* (LfAE) leaves and evaluated its in vivo antioxidant and anti-inflammatory potential.

**Methods:**

Polyphenols present in LfAE were characterized by high performance liquid chromatography (HPLC). Anti-inflammatory activity was studied in an experimental model of zymosan-induced intra-articular inflammation, conducted in Wistar rats treated with LfAE at the doses of 100, 200 and 300 mg/kg by gavage. Synovial fluid was collected for global leukocyte count, for spectrocopical UV/VIS analysis of myeloperoxidase (MPO) activity, total glutathione and malondialdehyde (MDA), and for quantification of inflammatory cytokines IL1-β and TNF-α by enzyme-linked immunosorbent assay. Synovial membrane was collected for histological analysis. The level of statistical significance was *p* < 0.05.

**Results:**

HPLC detected concentrations of 1.56 (0.77) %m/m for ellagic acid and 1.20 (1.38) %m/m for gallic acid in LfAE leaves. Treatment with LfAE at all doses significantly decreased the leukocyte influx into the synovial fluid (*p* < 0.001) and myeloperoxidase activity (*p* < 0.001), an important marker of neutrophils. LfAE at doses of 100 (*p* < 0.05), 200 and 300 mg/kg (*p* < 0.001) also reduced the levels of MDA. LfAE at doses of 200 and 300 mg/kg significantly decreased the levels of IL-1β (*p* < 0.05) and TNF-α (*p* < 0.001). All doses of LfAE resulted in increased levels of total glutathione (*p* < 0.001). Histopathological findings confirmed a reduction of the inflammatory infiltrate in the rats treated with LfAE at a dose of 200 mg/kg (*p* < 0.05).

**Conclusion:**

LfAE has an important anti-oxidant and anti-inflammatory effect on intra-articular inflammation.

## Background

Medicinal plants have been used by the population as an alternative treatment to several illnesses for thousands of years. In current times, the replacement of conventional therapy by new drugs based on natural products with antitumor, antimicrobial, anti-inflammatory and analgesic properties, and fewer adverse effects is still urgently needed [1, 2]. In researches focusing on the development of new drugs, the interest in plants has increased significantly worldwide [2].

*Libidibia ferrea (L. ferrea)*, popularly known in Brazil as *jucá* or *pau-ferro*, is a species of the Leguminosae family, one of the largest families among dicotyledons [3]. *L. ferrea* is found throughout the Northeast region of Brazil, where it is commonly used in folk medicine [4]. Folk medicine comprises medical aspects of traditional knowledge that developed over generations within various societies before the era of modern medicine [5]. In Brazil’s folk medicine, *L. ferrea* is widely used in treatment of many conditions due to its antidiarrheal, expectorant and healing activities [2, 6, 7]. Preliminary studies conducted with aqueous, acetonic and acetone-water extracts of *L. ferrea* have shown anti-inflammatory activity, reducing leukocyte migration in an animal peritonitis model, as well as antinociceptive activities [8, 9]. In addition, some compounds found in aqueous, acetonic and acetone-water extracts of *L. ferrea*, such as gallic acid catechin and ellagic acid, have been implicated in biological activities [9, 10].

Despite the anti-inflammatory activities of *L. ferrea* described in the literature, its potential therapeutic effects in rheumatoid arthritis (RA) remains unexplored. It is well-known that synovial inflammation plays a critical role in the symptoms and structural progression of osteoarthritis. In the RA joint microenvironment, synovial cells produce inflammatory mediators (cytokines and chemokines), activate chondrocytes, and propagate cartilage breakdown [11].

The presence of condensed tannins (catechins) and hydrolysable tannins (gallic acid) were found in the bark *of L. ferrea*. The bark of *L. ferrea* has also shown in vitro anti-inflammatory activity in a leukocyte migration model, and analgesic activity in the acetic acid-induced abdominal writhing test [9]. As for the Lipidic Portion of *L. ferrea*, the main components are capric acid, palmitic acid, palmitoleic acid, stearic acid, oleic acid, linoleic acid and linolenic acid, which presented an important antinociceptive effect in animal model experiments. This antinociceptive activity is possibly related to the *L. ferrea* ability to inhibit opioid, cholinergic receptors, and cyclooxygenase-2 pathway [8].

Considering the use of *L. ferrea* in folk medicine, further studies are needed to clarify its pharmacological properties. This study was designed to perform a phytochemical characterization of the *L. ferrea* crude extract, and to evaluate its effects on the intra-articular inflammation in a RA experimental model.

## Methods

### Plant material

Plant material constituted of *Libidibia ferrea* (Mart. ex. Tul.) L.P. Queiroz leaves was collected in the Caatinga biome of the city of Recife, Pernambuco – Brazil (8o3’ 30”S 34o54’ 12” W), in September 2014. After collection, it was dried in a circulating air oven (at 45 °C for 7 days). The material was identified by Dr. Rita de Cássia Pereira and then a voucher specimen was deposited at the herbarium of the Pernambuco Agronomic Institute (Instituto Agronômico de Pernambuco - IPA) under number 89419.

### Obtaining crude aqueous extract of *Libidibia ferrea* (Mart. ex. Tul.) L.P. Queiroz leaves (LfAE)

Crude extract of Libidibia ferrea (Mart. ex. Tul.) L.P. Queiroz leaves were obtained at 10% (*w*/*v*) by turbo extraction (four extractive cycles of 30 s, with 5 min of pause), using water as solvent [12]. The crude aqueous extract of *Libidibia ferrea * (Mart. ex. Tul.) L.P. Queiroz leaves (LfAE) was frozen at − 80 °C for three days and then lyophilized (Model L101, Liotop^®^). The quantification of ellagic acid and gallic acid was carried out by high performance liquid chromatography (HPLC) according to the methodology previously described by De Araújo et al. [9] and Guerra et al. [12] with an adaptation for total time (31 min).

### Animals

Male Wistar rats (*Rattus norvegicus albinus*, 150–250 g) were obtained from the laboratory of the Federal University of Rio Grande do Norte, Department of Biophysics and Pharmacology. The animals were kept in a room with a controlled temperature of 23 °C ± 2 °C in 12 h light-dark cycle, with access to food and water ad libitum. All efforts were made to minimize the number of animals used and any possible discomfort to them. Management of animal use followed the principles and guidelines approved by the Guide for the Care and Use of Laboratory Animals, while euthanasia followed the CONCEA Euthanasia Practice Guidelines. The protocols used were previously approved by the Ethics Committee on Animal Use (CEUA) through Opinion 01/2015.

### Zymosan-induced arthritis model

The method was performed as described by Chaves et al. [13]. The animals were divided into six groups with six animals each: normal control (without zymosan and treated with saline 0.9%), zymosan control (with zymosan and treated with saline 0.9%), standard (diclofenac 100 mg/kg) and LfAE (with zymosan, treated with LfAE 100, 200 and 300 mg/kg). One hour before the arthritis induction process, the animals were treated with either saline, or diclofenac, or LfAE. Arthritis induction was performed by intra-articular injection of 1 mg of zymosan dissolved in 50 μL of 0.9% saline solution into the rat knee joint. There was induction of intra-articular inflammation in all groups, except for the normal control, which only received 50 μL of 0.9% intra-articularly saline solution. The animals were intraperitoneally euthanized after 24 h with a lethal dose of thiopental (90 mg/kg) associated with lidocaine (10 mg/ml). Synovial fluid was collected for analysis of cellular influx, determination of myeloperoxidase (MPO) activity, glutathione, malondialdehyde (MDA), and inflammatory cytokines. The synovial membrane was removed for histopathological analysis.

### Experimental outcomes

The behavior and deaths of animals were recorded during the experiment.

### Analysis of the cellular influx

Synovial fluid collection was performed for all groups by washing the joint cavity with sodium phosphate buffer solution (0.15 M PBS, pH 7.4) in 0.01 M EDTA. The volume of 0.2 ml of the buffer in the joint and the aspirated volume were placed in an Eppendorf tube. Washing samples were used for global leukocyte count in a Neubauer chamber diluted in turkey solution at a 1:20 ratio.

### Determination of myeloperoxidase activity

Determination of MPO enzyme activity was made according to the procedure performed by Krawisz, Sharon and Stenson [14]. This analysis is based on the detergency action of 0.5% Hexadecyltrimethylammonium bromide (HTAB) buffer in 0.05 M saline phosphate buffer - pH 6.0 (PBS), which facilitates the MPO release from the granules into neutrophils. The reaction of this enzyme with the chromogenic reagent composed of o-dianisidine dihydrochloride (0.167 mg/ml) in 0.05 M PBS-pH 6.0 buffer with 0.0005% *w*/*v* hydrogen peroxide forms a chromophore that is absorbed at 450 nm.

The joint wash intended for analysis had been preconditioned in an eppendorf tube and then frozen at − 80 °C. The sample was then thawed for beginning the analysis. The amount of HTAB for each sample was calculated at the ratio of 1:20 *v*/v by means of the conditioned joint wash volume. Next, 20 μL of articular lavage was added to 400 μL of HTAB in all samples, which were homogenized for about 10 s by vortexing and taken to the freezer and subjected to a triple freeze-thaw process for 1 day. After the last thawing, the wash was centrifuged at 7000 G for 10 min at a temperature of 4 °C. Next, on an enzyme-linked immunosorbent assay (ELISA) plate, 50 μL of the peroxidase standard dilution was added to the calibration curve, 50 μL of the HTAB buffer to the blank and 50 μL of the sample supernatant, all in duplicates. To each well, 150 μL of the chromogenic reagent was added and the absorbance at 450 nm was then measured by Spectrocopical UV/VIS analysis (Biotek®, São Paulo, Brazil) at 0- and 3-min time at 37 °C. The enzymatic activity of MPO was calculated by interpolating the absorbance results in a standard curve performed with human neutrophil enzyme and horseradish peroxidase. The results were expressed as U/joint fluid.

### Determination of total glutathione content

Determination of total glutathione content was performed according to the method described by Anderson [15]. The joint wash intended for analysis was preconditioned in an Eppendorf tube and then frozen at − 80 °C with a volume of trichloroacetic acid (TCA 5% *w*/*v* in distilled water) in the ratio of 1:20 *v*/v to avoid degradation of GSH by gamma-glutamyltranspeptidase. Next, a 20 μl of sample was used for 400 μl of TCA. For determining this content, the samples were thawed and taken to an automatic homogenizer for about 2 min in the cold. Then the samples were centrifuged at 2000 G for 5 min at 4 °C. Next, 100 μl of the sample was withdrawn into an Eppendorf tube, then 100 μl of aqueous solution of sodium phosphate and EDTA (143 mM PBS-EDTA-PBS and 6.3 mM EDTA, pH 7.5) were added, along with 700 μl of the solution of NADPH (0.289 mM β-NADPH in PBS-EDTA) and 100 μl of the DTNB solution (6 mM DTNB in ​​PBS-EDTA). The solutions were taken to a water bath for 5 min at 30 °C, then transferred to ELISA plate wells, and 25 μl of the enzyme solution (glutathione reductase in PBS-EDTA 266 IU/ml) was added to each well. The absorbance was determined at a wavelength of 412 nm by Spectrocopical UV/VIS analysis (Biotek®, São Paulo, Brazil) at 0- and 3-min time. Total glutathione content was calculated by standard curve interpolation and the results were expressed as nmol/ml.

### Determination of malondialdehyde content

The determination of MDA content was performed as previously described [16]. The joint wash intended for analysis was preconditioned in an eppendorf tube and frozen at − 80 °C. For the analysis, the samples were thawed and diluted with 20 mM Tris HCl-pH 7.4 buffer in a ratio of 1:5 *v*/v, and 20 μl of sample and 100 μl of the buffer were used. The samples were taken to an automatic homogenizer for approximately 15 s and then taken to centrifugation at 2500 G for 10 min at 4 °C. 100 μl of the sample, 250 μl of the chromogenic reagent (1-methyl-2-phenylindole 10.3 mM in 3:1 acetonitrile) and 75 μl of 37% hydrochloric acid (HCL) were placed in another Eppendorf tube, which was incubated in a water bath at 45 °C for 40 min. The samples were then taken back for centrifugation for 5 min at 2500 G and transferred to the ELISA plate wells. The absorbance was determined at a wavelength of 586 nm by Spectrocopical UV/VIS analysis (Biotek®, São Paulo, Brazil). MDA content was calculated by standard curve interpolation and the results were expressed as nmol/ml.

### Levels of IL-1β and TNF- α inflammatory cytokines

Samples of synovium were stored at − 70 °C until required for each assay. The synovium liquid was homogenised and processed as described by Safieh-Garabedian et al. [17]. Levels of IL-1β (detection range: 62.5–4000 pg/mL; sensibility or lower limit of detection: 12.5 ng/mL of recombinant mouse IL-1β), and TNF-α (detection range: 62.5–4000 pg/mL; sensibility or lower limit of detection: 50 ng/mL of recombinant mouse TNF-α) in the synovium samples were determined with a commercial ELISA kit (R&D Systems, Minneapolis, MN, *USA*). All samples were within the wavelength used in UV-VIS spectrophotometry absorbance measured at 490 nm (Biotek®, São Paulo, Brazil).

### Histopathological analysis

The synovial membrane was collected and fixed in 10% formaldehyde, after which the procedures were carried out by routine methods until inclusion in paraffin blocks. Dehydration and other treatment steps aimed at extracting water from tissues by submerging them in baths with increasing concentrations of 70% ethanol to absolute ethanol. This solvent was replaced with xylol making the tissues translucent. Finally, the fragments embedded in paraffin were added. The tissue sections were placed in rectangular molds, forming blocks that were subsequently sectioned by 4 μm microtome steel knives for hematoxylin-eosin staining [18]. Histological evaluation was performed by assigning the following scores [19]: 0 - Inflammatory cell infiltrate and edema absent, absence of giant cells and granuloma, areas of fibrosis, neovascularization, necrosis and hemorrhage; 1 - Discrete inflammatory cell infiltrate and edema, rare giant cells and granuloma, with areas of fibrosis, neovascularization and discrete hemorrhages; 2 - Intense inflammatory cell infiltrate and edema, presence of giant cells and granuloma, with areas of fibrosis, neovascularization, necrosis and extensive hemorrhages; 3 - Intense inflammatory cell infiltrate and edema, presence of large numbers of giant cells and granuloma, with large areas of fibrosis, neovascularization, necrosis and hemorrhage. An experienced pathologist blind to the treatment protocol performed the analyses.

### Statistical analysis

All experimental data were plotted as mean ± standard deviation (SD). The results were statistically evaluated through the analysis of variance (one-way ANOVA) and Tukey-Kramer post hoc test. The Kruskal-Wallis test and Dunn’s post-test were used to compare the medians in the histopathological analysis. The level of statistical significance was *p* < 0.05. Analysis of the results and construction of graphs were performed using GraphPad Prism version 5.04.

## Results

**Fig. 1:**
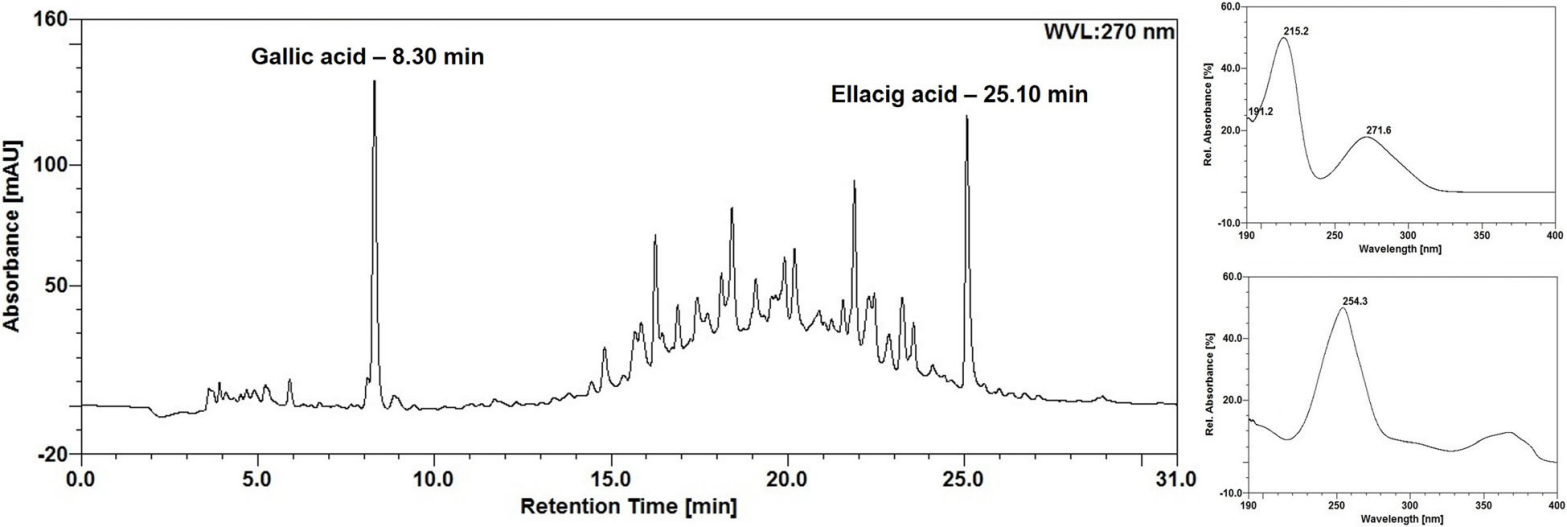
Chromatographic profile of LfAE and UV-spectrum of the standards (gallic acid - 8.30 min and ellagic acid - 25.1 min)

The total content of markers determined by HPLC in aqueous extract from leaves of *L. ferrea* was 1.56 (0.77) %m/m and 1.20 (1.38) %m/m for ellagic acid and gallic acid, respectively. Figure 1 demonstrates the chromatographic profile obtained for the extract and standards.

### Cellular influx

**Fig. 2:**
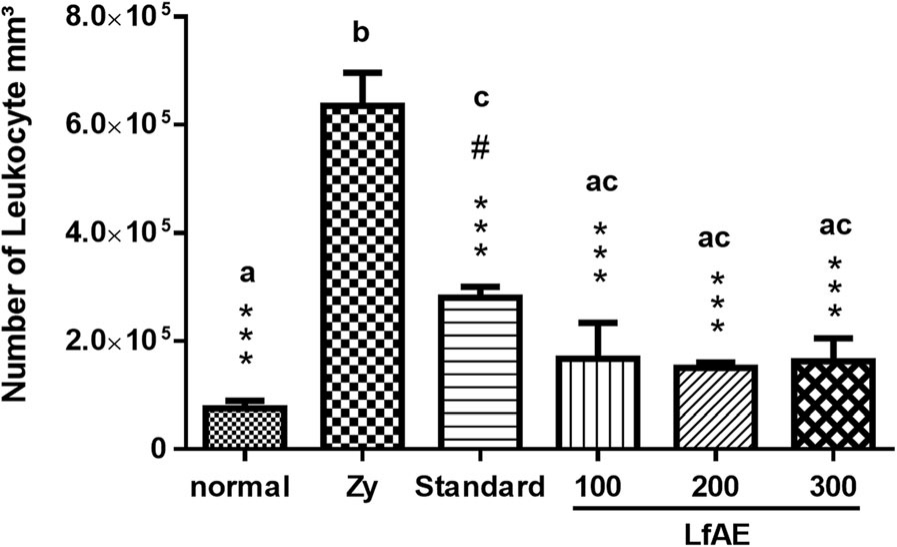
Effect of LfAE (100, 200 and 300 mg/kg) on cellular fluid influx in the zymosan-induced arthritis model (*n* = 6 per group). Data are expressed as mean ± SD (One-way ANOVA, Tukey-Kramer test, *n* = 6 per group). Letters (*a*, *b*, *c*) refer to the result of Tukey-Kramer post hoc test. Groups that do not share a letter have a mean difference that is statistically significant. ***(*p* < 0.001) vs zymosan group (Zy); #(*p* < 0.01) vs normal control group

Leukocyte cell infiltration into the synovial fluid was evaluated by the overall leukocyte count in the neubauer chamber. Figure 2 shows the effect of LfAE on the cellular influx. The extract at the doses of 100, 200 and 300 mg/kg reduced the leukocyte influx by 76 ± 2% (*p* < 0.001) compared to the zymosan control group. The standard group (diclofenac 100 mg/kg) reduced the leukocyte influx in the joint fluid by approximately 56% compared to the zymosan control group (*p* < 0.001).

### Determination of myeloperoxidase activity

**Fig. 3:**
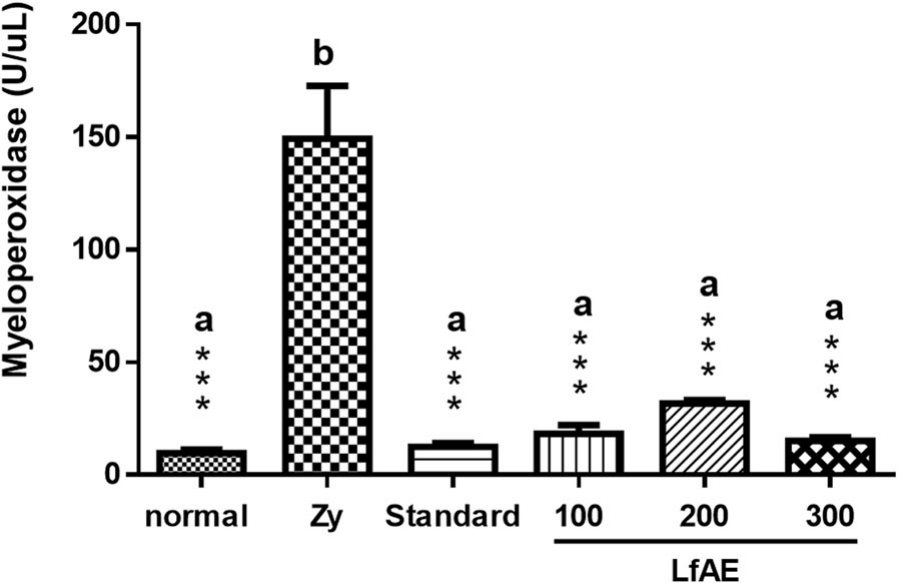
Effect of LfAE (100, 200 and 300 mgkg) on MPO levels in joint fluid in the zymosan-induced arthritis model. Data are expressed as mean ± SD (One-way ANOVA, Tukey-Kramer test, n = 6 per group). Letters (*a*, *b*) refer to the result of Tukey-Kramer post hoc test. Groups that do not share a letter have a mean difference that is statistically significant. ***(*p* < 0.001) vs zymosan control group (Zy)

Neutrophil influx results can be assessed by MPO activity in the joint fluid. LfAE at doses of 100, 200 and 300 mg/kg reduced MPO levels by approximately 85% ± 7% (*p* < 0.001) when compared to the zymosan control group, which presented a very high level of the enzyme (mean of 150 U/joint fluid). Animals treated with diclofenac (100 mg/kg) also showed a significant decrease in MPO levels in the rat’s joint fluid (*p* < 0.001) (Fig. 3).

### Determination of total glutathione content

Total glutathione content (GSH + GSSG) was determined to assess the integrity of the antioxidant defense system of the body. In this process, the extract showed a beneficial effect, demonstrated by the ability to prevent the reduction of total glutathione levels.

**Fig. 4:**
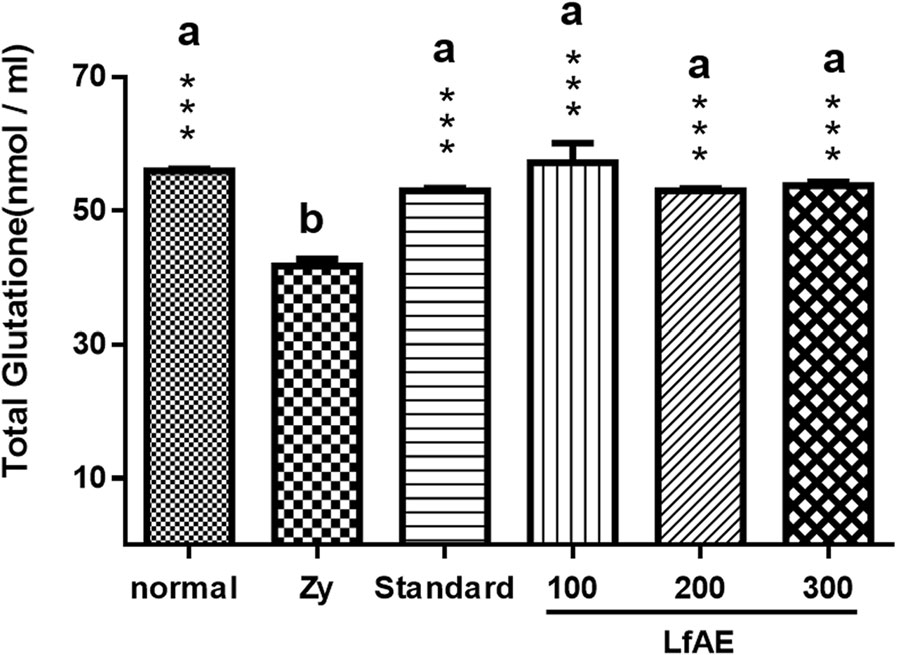
Effect of LfAE (100, 200 and 300 mg/kg) on total glutathione levels in the zymosan-induced arthritis model. Data are expressed as mean ± SD (One-way ANOVA, Tukey-Kramer test, *n* = 6 per group). Letters (*a*, *b*) refer to the result of Tukey-Kramer post hoc test. Groups that do not share a letter have a mean difference that is statistically significant. ***(*p* < 0.001) vs zymosan control group (Zy)

Zymosan control group exhibited a significant decrease in total glutathione levels (41 nmol/ml) compared to normal control group (healthy animals), which showed a mean of 56 nmol/ml of articular liquid (*p* < 0.001). Animals treated with LfAE at all doses were able to significantly prevent the reduction of total glutathione levels when compared to the zymosan control group (*p* < 0.001). The standard group (diclofenac 100 mg/kg) also showed important inhibition of the reduction of total glutathione levels (*p* < 0.001). The LfAE at a dose of 100, 200 and 300 mg/kg showed a similar preventive potential, demonstrating effectiveness in maintaining antioxidant protection (Fig. 4).

### Determination of malondialdehyde content

**Fig. 5:**
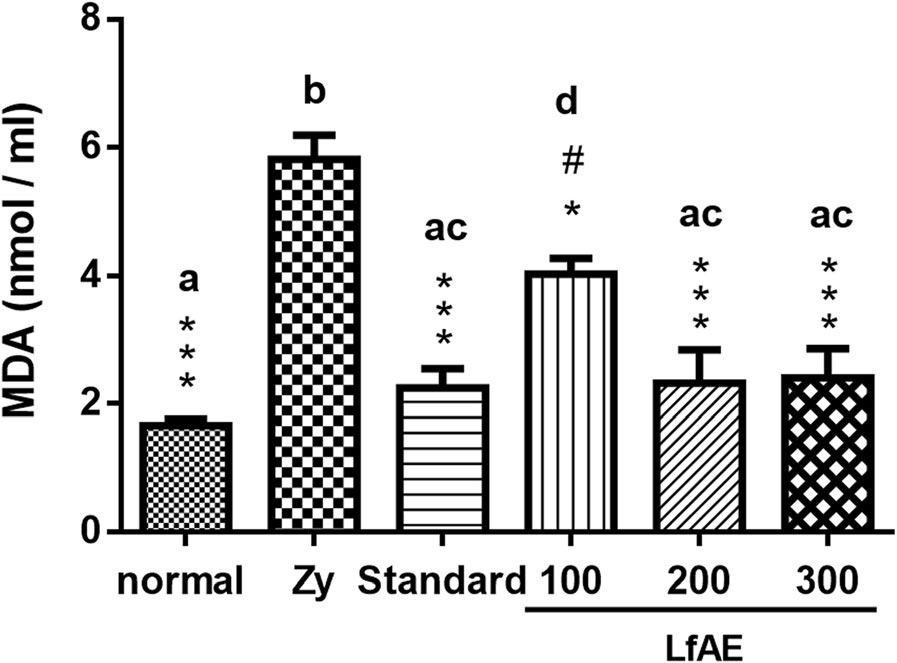
Effect of LfAE (100, 200 and 300 mg/kg) on the levels of malondialdehyde in the zymosan-induced arthritis model. Data are expressed as mean ± SD (One-way ANOVA, Tukey-Kramer test, *n* = 6 per group). Letters (*a*, *b*, *c*, *d*) refer to the result of Tukey-Kramer post hoc test. Groups that do not share a letter have a mean difference that is statistically significant. *(*p* < 0.05), ***(*p* < 0.001) vs zymosan control group (Zy); #(*p* < 0.05) vs normal control group

Another method to evaluate the antioxidant defense system is by determining the MDA content. The results showed that LfAE significantly reduced MDA levels at a dose of 100 mg/kg (*p* < 0.05) and 60% at doses of 200 and 300 mg/kg (*p* < 0.001) compared to the zymosan control, which presented a high concentration of MDA (6 nmol/ml of joint fluid). Animals treated with diclofenac 100 mg/kg (standard) revealed a significant reduction of MDA levels (approximately 62%) in the articular fluid, compared to the zymosan control group (*p* < 0.001). Similar results were obtained in rats treated with LfAE at 200 and 300 mg/kg doses (Fig. 5). LfAE at a dose of 100 mg/kg showed a significant difference when compared to the the zymosan control group (*p* < 0.05), as well as compared to the standard, and LfAE at 200 and 300 mg/kg doses (*p* < 0.001) (Fig. 5).

### Inflammatory cytokines

**Fig. 6:**
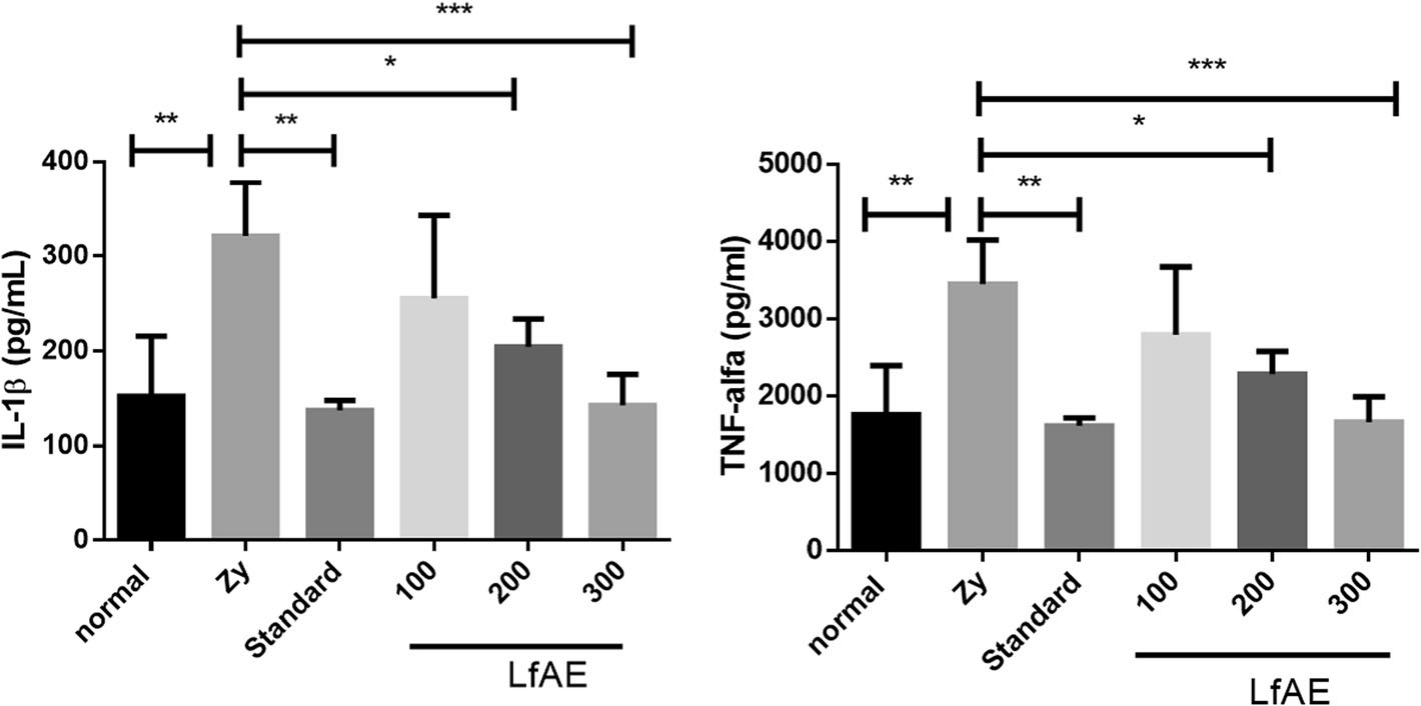
Effect of LfAE (100, 200 and 300 mg/kg) and Standard on IL-1β and TNF-α levels in the zymosan-induced arthritis model. Data are expressed as mean ± SD (One-way ANOVA, Tukey-Kramer test, *n* = 6 per group). *(*p* < 0.05); **(*p* < 0.01); ***(*p* < 0.001) vs zymosan control group (Zy)

A significant decrease of IL-1β levels was observed in the standard group (*p* < 0.01) and in the animals treated with LfAE at doses of 200 and 300 mg/kg (*p* < 0.05 and *p* < 0.001, respectively), when compared to zymosan control group (Fig. 6).

The results also showed a significant reduction of TNF-α in the standard group, treated with diclofenac 100 mg/kg (*p* < 0.01) and in the groups treated with LfAE at doses of 200 and 300 mg/kg (*p* < 0.05 and *p* < 0.001, respectively), in comparison to the zymosan control group (Fig. 6).

### Histopathological analysis of synovial membrane

**Fig. 7:**
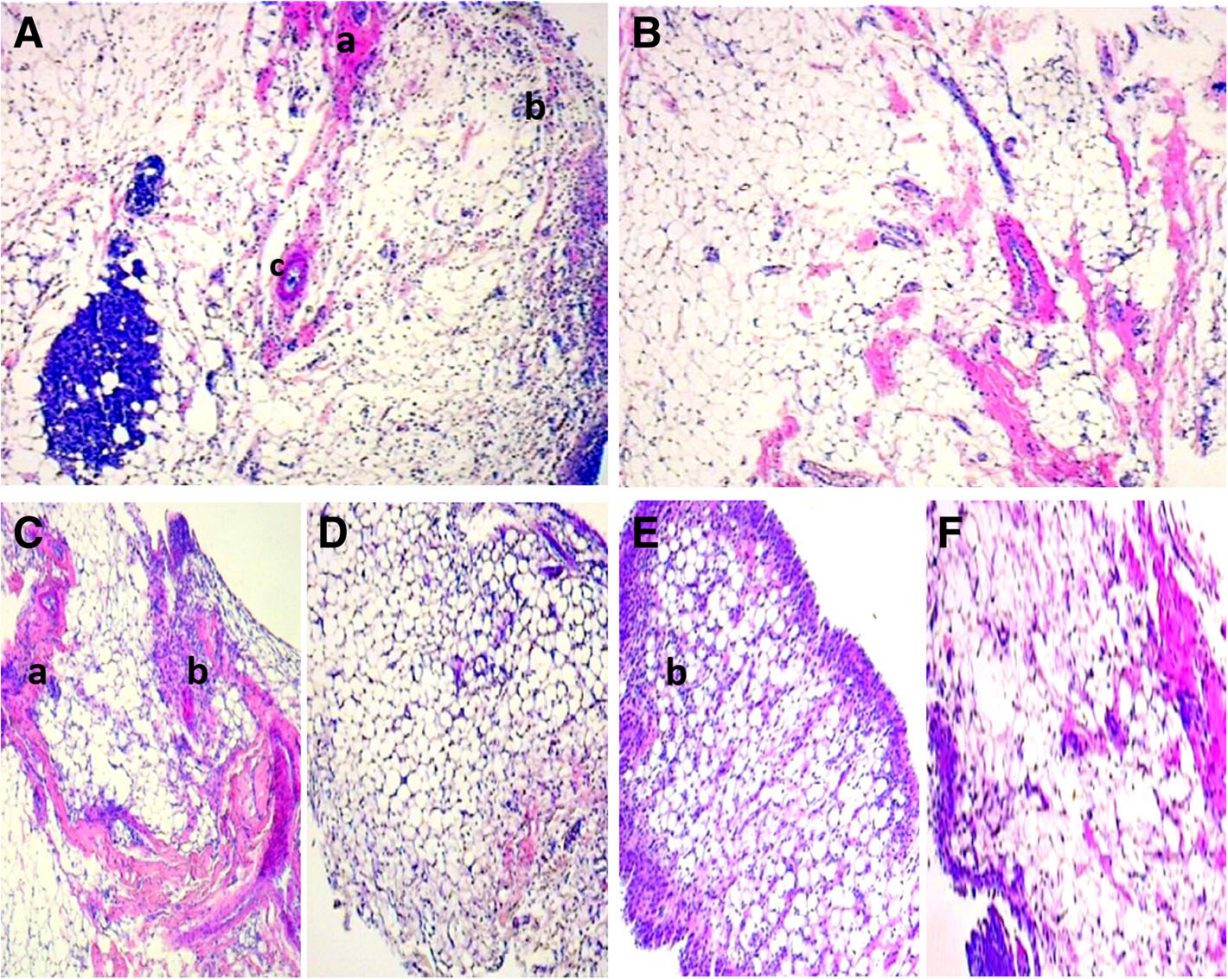
Microphotograph of H&E-stained histological section of the synovial membrane of rats (40x magnification). **a** zymosan control: intense leukocyte infiltrate with fibrosis area. **b** normal control: structures preserved and within normality. **c** LfAE 100 mg/kg with presence of leukocyte infiltrate and areas of fibrosis. **d** LfAE 200 mg/kg presenting reduction of inflammatory parameters in the synovial membrane. **e** LfAE 300 mg/kg group presenting mild leukocyte infiltrate in the synovium. **f** Standard 100 mg/kg with mild leukocyte infiltrate. *a* - fibrosis; *b* - inflammatory infiltrate; *c* – neovascularization

Results of the histopathological scores are presented in Table 1. Histopathological assessment of joints from untreated zymosan control animals (Figs. 7a) was characterized by marked leukocyte infiltration in the synovium, in addition to the presence fibrosis area and neovascularization (score 3). However, LfAE-treated rats had significantly decreased levels of cellular infiltration, and this improved histopathology was not dose-dependent. The animals of the normal control group (Fig. 7b) had no abnormalities or inflammatory reaction, hence the parameters of the histopathological analysis were absent (score 0).

**Table 1:** LfAE effect (100, 200 and 300 mg/kg) on the histopathological scores according to the experimental groups

Group	Score
Zymosan Control	3 (3–3) ***
Normal Control	0 (0–0) ***
Standard/Diclofenac 100 mg/kg	1 (1–1) ***
LfAE 100 mg/kg	2 (2–3) ***
LfAE 200 mg/kg	1 (1–2) ***
LfAE 300 mg/kg	2 (1–3) ***

*(*p* < 0.05); **(*p* < 0.01); ***(*p* < 0.001) vs zymosan control (*Kruskal-Wallis, Dunn’s*). (*n = 6*/group)

*L. ferrea* aqueous extract reduced the intensity of leukocyte cell infiltrate and other parameters at doses of 100 mg/kg (score 2), 200 mg/kg (score 1) and 300 mg/kg (score 2), according to Fig. 7c, d and e, with a statistically significant difference only at the dose of 200 mg/kg compared to the zymosan control group. Thus, the results show that LfAE carried out an important anti-inflammatory effect on the installed inflammatory process, and the dose of 200 mg/kg achieved the best performance. Analysis of synovium from the diclofenac-treated group/standard (Fig. 7f) showed a reduction in inflammatory parameters (score 1) when compared to the positive zymosan control group, revealing that the drug exerted the desired anti-inflammatory activity.

## Discussion

RA is an autoimmune disease with a complex pathophysiology and limited therapeutic alternatives. It is characterized by joint edema, movement-induced joint hyperalgesia and inflammatory cell infiltration. Arthritis animal model, such as the zymosan-induced model, is characterized by synovial inflammation, cartilage destruction and bone erosion, and is considered an appropriate representation of human RA pathogenic processes. Zymosan is a polysaccharide from yeast cell walls of Sarccharomyces cerevisiae composed of β-1,3-glycosidic bonds [20]. It is composed of proteins, chitin, mannan, lipids and β-glucan (its main active properties) [21]. When zymosan is injected into rat knee joints, it produces severe and erosive synovitis, which is also associated with significant leukocyte influx, mainly due to neutrophil accumulation in the knee synovial washes [22].

*Libidibia ferrea* (Mart. ex Tul.) L.P. Queiroz is a tree native to Brazil. Various parts of this species (bark, fruits, leaves, and seeds) have been used in Brazilian folk medicine due to their many pharmacological properties [10, 20]. The Association of Community Health Agents of Igarapé in partnership with the local nucleus of *Pastoral da Criança* (Pará, Brazil) has informally produced folk medicinal plant preparations to serve the local population for some years [21].

In this sense, several biological properties are reported for the aqueous crude extract of the fruits of *L. ferrea*, such as anti-inflammatory and analgesic effects [22]. Extracts and polysaccharide fractions of the *L. ferrea* pods have also demonstrated anti-inflammatory activity [23]. However, there are no scientific reports in Brazil’s folk medicine regarding the effects of *L. ferrea* specifically on RA.

The present results demonstrated that aqueous extract from leaves of *L. ferrea* had 1.56 and 1.20% of ellagic acid and gallic acid, respectively. Ellagic acid is present in the plant vacuole and has an antioxidant and anti-inflammatory effect [12]. According to previous reports, ellagic acid inhibits lipid peroxidation induced by γ-radiation (hydroxyl radical) in rat liver microsomes [24]. The gallic acid is a phenolic compound constitutively present in a wide variety of fruits and plants [12]. The radical scavenging efficiency of the gallic acid derivatives is dependent on the presence of hydroxyl groups, as well as steric freedom. In particular, the para-substituted –OH group was found to be highly efficient in radical scavenging. The extra hydroxyl group in trihydroxy phenolic acid provides greater stability and higher antioxidant activity. The –OH group ortho to phenol tends to stabilize the radical formed, resulting in a lower hydrogen bond dissociation enthalpy and hence, an increased antioxidant capacity [25].

The inflammatory joint process induced by zymosan, similarly to patients with RA, is characterized by the presence of oxidative stress that contributes to joint damage. The overproduction of reactive oxygen and nitrogen species during oxidative stress causes damage to the polyunsaturated fatty acids of the cellular lipid membrane. Subsequently, the lipid peroxidation process is initiated, which changes the structural and functional organization of the cell and generates products, such as MDA [26]. Analysis of MDA is used as an evaluation parameter for lipid peroxidation in inflammatory processes, and consequently, as an indicator of free radical activity in the body. Elevated levels of MDA have been reported in patients with RA [27].

We observed that all doses of LfAE were effective in reducing MDA levels in the rat’s joint fluid, maintaining antioxidant protection. In vitro experiments demonstrated that crude extract of the fruits of L. ferrea was able to significantly reduce MDA levels in colorectal cancer, reinforcing the antioxidant potential of this plant [12].

The damage caused by oxidative stress can also be evaluated by analyzing the glutathione antioxidant. The catalytic cycle of glutathione formed by the glutathione oxidase, glutathione peroxidase and glutathione reductase enzymes guarantees the protection of the body against oxidative radicals. The determination of total glutathione aims to evaluate the integrity of this antioxidant system in biological tissues and fluids. Therefore, it is expected that the greater the oxidative stress at the inflammatory site, the lower the antioxidant protection system. We observed that LfAE at any dose level exhibited a significant protective role against glutathione depletion. Taken togheter, these results suggest that the LfAE prevented oxidative stress in the rats joints, which corroborate the antioxidant potential of the *L. ferrea* reported in the literature [28].

The pathophysiology of RA is related to the recruitment of T cells and self-reactive leukocytes in the joint, representing a major contributory factor to the excessive production of inflammatory mediators by resident and/or infiltrating inflammatory cells. The leukocyte infiltrate, mainly neutrophils, induces the release of MPO at the inflammatory site [29, 30].

In the present study, MPO analysis was performed to confirm the results obtained by the leukocyte influx analysis. These data were confirmed by the joint histopathological assessment. We verified that LfAE presented a significant reduction of MPO levels in the articular fluid, similar to the effect observed when the standard drug (diclofenac) was used. However, this effect was not dose-dependent, which may be related to the dose range selected that may not be enough to denote a significant difference in the response.

Histopathological assessment of the synovial membrane from the zymosan control group was characterized by marked leukocyte infiltration in the synovium, in addition to the presence of fibrosis and neovascularization. The microscopic features confirmed that LfAE reduced the intensity of leukocyte cell infiltrate with a statistically significant difference at the dose of 200 mg/kg when compared to the zymosan control group. Araujo et al. [9] described the aqueous and acetone-water extracts of L. ferrea as significant inhibitors of leukocyte migration in vivo.

Leukocytes are responsible for the production of various inflammatory mediators such as cytokines, chemokines and matrix metalloproteinases, that cause cartilage and bone destruction. Among the cytokines involved in RAinflammatory process, there are TNF-α, IL-1, IL-6, IL-10, IL-17 and NF-kB [31]. Specifically, TNF-α and IL-1 can mediate immune functions that induce inflammatory response and tissue formation, leading to cartilage destruction and bone erosion in RA [32].

We observed a significant reduction of proinflammatory cytokines (TNF-α, IL-1β) in the joints of rats treated by LfAE at doses of 200 and 300 mg/kg. According to previous reports, gallic acid can suppress the release of TNF-α by neutrophis, which appear to be related to its inhibitory effect on cAMP-phosphodiesterase 4 activity [33]. Additionaly, Ahad et al. [34] suggested that ellagic acid can suppress the synthesis of IL-1β and TNF-α by inhibiting the NF-κB pathway.

In this study, we have shown that oral administration of LfAE has a beneficial effect on the severity of arthritic inflammation. The substantial changes observed after treatment with LfAE included the reduction of proinflammatory cytokines (TNF-α, IL-1β) and oxidative stress markers (MDA, glutathione) in the joint tissues.

## Conclusion

LfAE presented an important anti-inflammatory activity in the zymosan-induced arthritis model. Oral treatment with LfAE was able to inhibit synovial inflammation, decrease the leukocyte infiltrate, MPO activity and MDA levels.It also prevented a reduction of total glutathione levels and improved the histopathological analysis parameters. In addition, LfAE reduced levels of inflammatory markers IL-1β and TNF-α in the joint tissue. These data emphasize the importance of natural products in seeking new bioactive compounds and highlight the anti-inflammatory potential of *Libidibia ferrea* extract, which may contribute to the development of herbal medicine, while confirming its ethnopharmacological use in treating inflammatory processes**.**

## Abbreviations

AP-1: Protein 1 activator
AR: Rheumatoid arthritis
CCD-AE: High Efficiency thin layer chromatography
CEUA: Commission on ethics in animal use
CLAE: High Efficiency liquid chromatography
CONCEA: National council for control of animal experimentation
DTNB: Dithiobisnitrobenzoic acid
EDTA: Ethylenediamine tetraacetic acid
ERO: Reactive oxygen species
FR: Rheumatoid factor
GSH: Reduced glutathione
GSSG: Oxidized glutathione
HCL: Hydrochloric Acid
HTAB: Hexadecyltrimethylammonium bromide
IgG: Immunoglobulin G
IgM: Immunoglobulin M
IL: Interleukin
LfAE: Aqueous extract of libidibia ferrea
LNRP: Recall nociceptive threshold
MDA: Malondialdehyde
MPO: Myeloperoxidase
NADPH: Nicotinamide adenine dinucleotide reduced phosphate
NF-kB: Nuclear Kappa B factor
PBS: Capped sodium cap
TCA: Trichloroacetic acid
TNF-α: Tumor necrosis factor alpha
